# Cross-Reactive T Cells Are Involved in Rapid Clearance of 2009 Pandemic H1N1 Influenza Virus in Nonhuman Primates

**DOI:** 10.1371/journal.ppat.1002381

**Published:** 2011-11-10

**Authors:** Jason T. Weinfurter, Kevin Brunner, Saverio V. Capuano, Chengjun Li, Karl W. Broman, Yoshihiro Kawaoka, Thomas C. Friedrich

**Affiliations:** 1 Wisconsin National Primate Research Center, Madison, Wisconsin, United States of America; 2 Department of Pathobiological Sciences, University of Wisconsin School of Veterinary Medicine, Madison, Wisconsin, United States of America; 3 Department of Biostatistics and Medical Informatics, University of Wisconsin School of Medicine and Public Health, Madison, Wisconsin, United States of America; 4 Division of Virology, Department of Microbiology and Immunology, Institute of Medical Science, University of Tokyo, Tokyo, Japan; 5 Department of Special Pathogens, International Research Center for Infectious Diseases, Institute of Medical Science, University of Tokyo, Tokyo, Japan; 6 ERATO Infection-Induced Host Responses Project, Saitama, Japan; Oregon Health and Science University, United States of America

## Abstract

In mouse models of influenza, T cells can confer broad protection against multiple viral subtypes when antibodies raised against a single subtype fail to do so. However, the role of T cells in protecting humans against influenza remains unclear. Here we employ a translational nonhuman primate model to show that cross-reactive T cell responses play an important role in early clearance of infection with 2009 pandemic H1N1 influenza virus (H1N1pdm). To “prime” cellular immunity, we first infected 5 rhesus macaques with a seasonal human H1N1 isolate. These animals made detectable cellular and antibody responses against the seasonal H1N1 isolate but had no neutralizing antibodies against H1N1pdm. Four months later, we challenged the 5 “primed” animals and 7 naive controls with H1N1pdm. In naive animals, CD8+ T cells with an activated phenotype (Ki-67+ CD38+) appeared in blood and lung 5–7 days post inoculation (p.i.) with H1N1pdm and reached peak magnitude 7–10 days p.i. In contrast, activated T cells were recruited to the lung as early as 2 days p.i. in “primed” animals, and reached peak frequencies in blood and lung 4–7 days p.i. Interferon (IFN)-γ Elispot and intracellular cytokine staining assays showed that the virus-specific response peaked earlier and reached a higher magnitude in “primed” animals than in naive animals. This response involved both CD4+ and CD8+ T cells. Strikingly, “primed” animals cleared H1N1pdm infection significantly earlier from the upper and lower respiratory tract than the naive animals did, and before the appearance of H1N1pdm-specific neutralizing antibodies. Together, our results suggest that cross-reactive T cell responses can mediate early clearance of an antigenically novel influenza virus in primates. Vaccines capable of inducing such cross-reactive T cells may help protect humans against severe disease caused by newly emerging pandemic influenza viruses.

## Author Summary

Antibodies against influenza target the highly mutable proteins on the virus surface. Influenza pandemics are caused by novel viruses whose surface proteins are so different from previously circulating viruses as to be unrecognizable by most individuals' antibodies. We hypothesized that T cells might be capable of reducing the severity of infection with pandemic influenza viruses, against which antibodies are ineffective. Experiments in mice have supported this idea, but the ability of T cells to protect humans against influenza has remained unclear. We therefore tested our hypothesis in macaque monkeys, whose physiology and immune systems closely resemble those of humans. We used a seasonal virus to “prime” macaques to make immune responses against influenza and found that these animals were able to control infection with 2009 H1N1 pandemic influenza viruses more effectively than animals that had not been “primed.” Protection was associated with T cell responses, but not antibodies, that were quickly “recalled” after challenge with the pandemic virus. Our results suggest that “cross-reactive” T cells could play an important role in controlling influenza in humans. Vaccines designed to induce strong T cell responses in addition to antibodies could offer enhanced protection against emerging influenza viruses.

## Introduction

The emergence and rapid spread of a novel triple reassortant H1N1 influenza virus in April 2009 raised fears of a new and severe pandemic. Early reports from Mexico suggested that the 2009 virus might be more pathogenic than typical seasonal strains [1]. It was quickly determined that the virus's hemagglutinin (HA) envelope protein was derived from the “classical swine” lineage, and therefore descended from the H1N1 virus that emerged in 1918 [2]. More troublingly, few individuals under age 65 appeared to have antibodies capable of recognizing the emerging virus [3]–[5]. By June 2009 the World Health Organization had declared the first influenza pandemic of the 21st Century. At the same time, manufacturers and health ministries raced to produce, approve and deploy vaccines that could protect against the 2009 H1N1 pandemic virus (H1N1pdm) [6].

When pre-existing antibody responses are insufficient for protection, T cell responses that recognize relatively well-conserved peptide epitopes may play an important role in promoting viral clearance and reducing influenza disease severity. In mice CD8+ T cells primed by influenza viruses of one subtype can mediate “heterosubtypic immunity” against challenge with serologically distinct viruses in the absence of cross-reactive antibody [7]–[11]. Accordingly, a recent study in ferrets suggested that previous infection with seasonal influenza viruses reduced shedding of H1N1pdm [12]. Similar serial infection studies in mice suggest that cross-reactive T cells raised by infection with seasonal influenza strains can protect against lethal challenge with H1N1pdm, while antibodies cannot [13]. But the role of T cells in human immunity to influenza remains unclear. A retrospective epidemiological study suggested that cellular immune responses induced by recent infection might have protected individuals from severe disease in the 1957 pandemic [14]. Similarly, cytotoxic T lymphocyte activity was associated with reduced virus shedding in a cohort of experimentally infected volunteers who had no detectable antibody responses against the experimental strain [15]. More recently, in-vitro studies have shown that T cells from human volunteers cross-react with peptide epitopes that are conserved among distinct viral subtypes [16]–[19]. Furthermore, in a prospective study of influenza vaccine effectiveness, the presence of virus-specific T cell responses reduced elderly subjects' risk of developing clinically apparent influenza, while there was no correlation between vaccine-induced antibody titers and influenza risk [20]. Despite these findings, however, there is little direct evidence from humans that T cells mediate protection from influenza disease or enable rapid virus clearance.

We therefore developed a translational model of immunity to influenza using rhesus macaques, which share a closer phylogenetic relationship with humans than mice or ferrets do. Macaques have been used previously for influenza research, but T cell responses have rarely been examined [21]–[29]. Interestingly, however, one study of DNA-based vaccines in macaques suggested that T cells alone might be capable of limiting replication of H5N1 viruses and protecting vaccinated animals against severe disease, while vaccine strategies that induced both T cells and neutralizing antibodies appeared to be even more effective [30]. In the present study, we asked whether T cells “primed” by infection with a seasonal human influenza virus would enable macaques to rapidly clear subsequent challenge with H1N1pdm. We found evidence that such cross-reactive T cell responses are indeed elicited by infection with seasonal viruses in these animals and may serve to limit virus replication after challenge with a heterologous 2009 pandemic isolate. Our results suggest that vaccines capable of stimulating T cell responses could play an important role in mitigating the severity of future influenza pandemics, when pre-existing antibodies are not likely to be effective. The only currently accepted correlate of vaccine-induced protection against influenza is serum neutralizing antibody. We speculate that vaccine components designed to induce strong cross-reactive T cell responses in addition to antibodies may augment currently approved vaccine modalities.

## Results

### A seasonal human influenza virus replicates in macaques and induces detectable cellular immune responses

**Figure 1:**
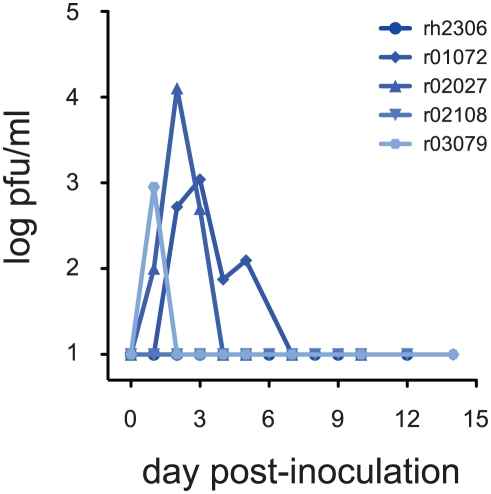
A seasonal human influenza virus replicates in rhesus macaques. Five rhesus macaques were inoculated with 9 million plaque-forming units (pfu) influenza virus A/Kawasaki/173/2001 (K173, H1N1) by a combination of routes as described in the text. Animals had no detectable antibodies against K173 at the time of inoculation. Virus replication in nasal secretions was monitored using standard plaque assays. Virus titer is expressed as pfu/ml nasal swab fluid.

To model exposure of humans to seasonal influenza viruses prior to the 2009 pandemic, we first inoculated 5 rhesus macaques with a recent seasonal isolate, A/Kawasaki/173/2001 (K173, H1N1). Hemagglutination-inhibition (HI) assays using serum from these animals confirmed that they had no pre-existing neutralizing antibody responses against K173 **(Table 1)**. This isolate had productively infected cynomolgus macaques in a previous study [29]. Animals were inoculated with a total of 9 million pfu virus by a combination of routes (nasal, ocular, tracheal), as described previously [31]. After inoculation we detected infectious virus in nasal secretions from 3 of the 5 animals **(Fig. 1)**. Virus was also isolated from tracheal secretions in a fourth animal, r02108 (data not shown). Virus replication in nasal secretions peaked between 1 and 4 days after inoculation, reaching peak titers between 900 and 12,750 pfu/ml **(Fig. 1)**, consistent with the kinetics and magnitude of seasonal influenza virus replication in experimentally inoculated humans [32]. We did not detect infectious K173 in bronchoalveolar lavage (BAL) samples from any of the 5 animals (data not shown), suggesting that this seasonal virus did not replicate in the lower respiratory tract.

**Table 1:** Serum antibody titers against seasonal virus K173.

	K173-specific HI^a^			
animal	K173 prechallenge	day 28 post-K173	H1N1pdm prechallenge	day 21 post-H1N1pdm
rh2306	<10^b^	320	128	320
r01072	<10	64	64	320
r02027	<10	64	64	80
r02108	<10	40	64	160
r03079	<10	64	64	40
r02002	na^c^	Na	<8	<10
r03087	na	Na	<8	<10
r03089	na	Na	<10	<10
r03137	na	Na	<10	<10
r04052	na	Na	<10	<10
r04077	na	Na	<10	<10
r05092	na	Na	<10	<10

a serum HI titers against seasonal virus A/Kawasaki/173/2001 ^b^HI assays conducted using turkey red blood cells and animal serum with seasonal virus K173. Detection limit 8 or 10, depending on lowest serum concentration tested. ^c^na, not applicable: naive animals were not challenged with seasonal virus K173.

**Figure 2:**
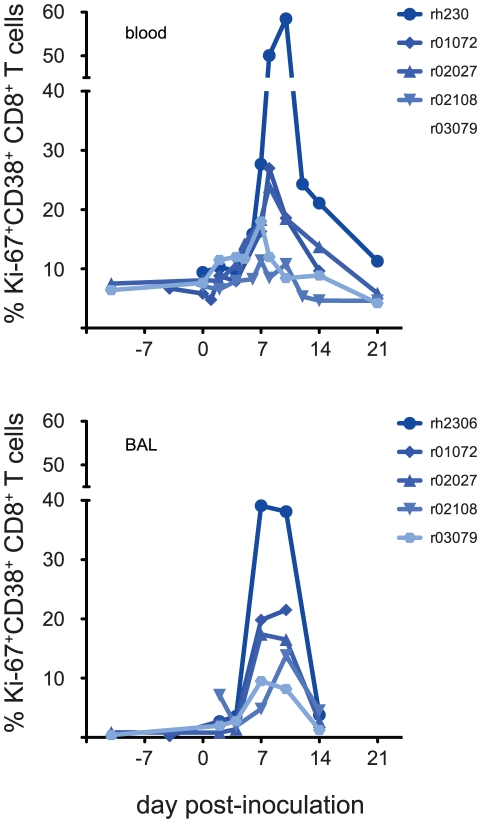
Activated CD8+ T cells appear in blood and lung within 7 days of inoculation with seasonal influenza virus K173. We used a flow cytometric assay to enumerate CD8+ T cells with an activated phenotype before and after K173 infection as described in the text. Data plotted are the frequency of CD3^+^ CD8^+^ lymphocytes that express both Ki-67 and CD38. Data are not available from animal r01072 at day 21 post-inoculation due to technical problems.

No influenza-derived CD8+ or CD4+ T cell epitopes have been reported for infected macaques, so we could not use epitope-specific reagents such as minimal optimal peptides or MHC tetrameric complexes to measure cellular immune responses in these animals. Therefore, to track induction of cellular immunity against K173 we first measured activation of T cells in blood and lung following virus inoculation. In acute viral infections, “activated” T cells express the proliferation marker Ki-67 and high levels of the adhesion molecule CD38 [33]. HLA-DR was not consistently upregulated on Ki-67+CD38+ T cells in our experiments **(Fig. S1)**. Expanding populations of CD8+ T cells expressing this phenotype were detectable both in the periphery (blood) and in the lung (BAL) within 7 days of inoculation in all 5 animals **(Fig. 2)**. We did not directly assess the activation kinetics of CD4+ T cells, but we can estimate the frequency of activated CD4+ T cells using cells that express the T cell marker CD3, but not CD8 **(Fig. S2)**. Populations of activated CD3+CD8- T cells also expanded in both compartments with similar kinetics, though the frequency of activated CD8- T cells was much lower than that of activated CD8+ T cells, particularly in the blood; the sole exception to this trend was animal rh2306, in which 53.8% of CD3+CD8- cells in BAL had an activated phenotype at day 7 **(Fig. S3)**. Indeed, the highest levels of CD8+ T cell activation in lung and blood were also observed in animal rh2306, the only animal from which infectious K173 virus could not be isolated. 58.5% of this animal's blood CD8+ T cells and 39.1% of its lung CD8+ T cells were activated at the peak of its response in these compartments, on days 7–10 post-infection.

**Figure 3:**
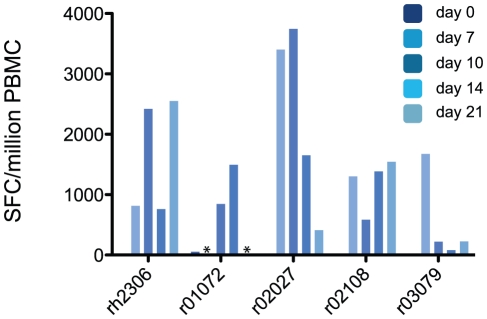
Macaques mount virus-specific T cell responses after K173 infection. We used an IFN-γ Elispot assay to enumerate influenza virus-specific T cells among the peripheral blood mononuclear cells (PBMC) of infected macaques. PBMC were stimulated with 19 pools of overlapping synthetic peptides that collectively represented the influenza virus proteome, as described in the text. After subtracting background signal from the results for each pool, the frequency of cells responding to each pool were summed to give the total virus-specific response magnitude. Results are expressed as the number of spot-forming cells (SFC) per million PBMC. To be considered positive, the frequency of peptide-specific SFC/million PBMC must be at least 3 times larger than the background (no peptide) value. Asterisks indicate that data are not available from animal r01072 from days 7 or 21 post-inoculation due to technical problems.

To confirm that these expanding populations of activated T cells represented an influenza virus-specific cellular immune response, we performed interferon (IFN)-γ Elispot assays using a library of overlapping peptides representing the entire influenza virus A (H1N1) proteome, with the exception of PB1F2. The peptides in this library have between 99% and 100% amino acid sequence identity with K173 proteins (See **Table S1**). The sum of virus-specific T cell responses detected by Elispot at each timepoint is shown in **Fig. 3**. Importantly, we detected no cells secreting IFN-γ in response to stimulation with influenza peptides prior to infection in any animal. However, each animal made detectable T cell responses against influenza peptides on at least two timepoints after inoculation with K173. The total magnitude of the virus-specific T cell response was greater than 1,000 spot-forming cells (SFC) per million PBMC at one or more timepoints in each animal, with peak magnitudes ranging from 1,495 to 3,745 SFC/million. Responses to a peptide pool representing the N-terminal half of NP were immunodominant in all 5 animals **(Fig. S4)**. To determine the contribution of CD8+ T cells to the total peptide-specific response, we repeated Elispot assays on cryopreserved PBMC from day 7 post-infection using 2 peptide pools that stimulated strong responses in each animal. In these assays we determined the frequency of IFN-γ-secreting cells in both whole PBMC and in PBMC depleted of CD8+ cells. CD8+ cell depletion diminished or abolished peptide-specific cytokine secretion in response to many pools, but for most animals at least one peptide pool elicited IFN-γ responses in both whole PBMC and the depleted cell fraction (data not shown). Intracellular cytokine staining (ICS) of peptide-stimulated PBMC allows the simultaneous and direct enumeration of peptide-specific CD4+ and CD8+ T cells. ICS assays on cryopreserved PBMC using selected peptide pools confirmed that, as a group, animals made both CD4+ and CD8+ T cell responses against influenza virus antigens **(Table S2)**. Together, these data show that the seasonal human influenza virus K173 can productively infect rhesus macaques and that this infection induces detectable virus-specific cellular immune responses that involve both CD4+ and CD8+ T cells, with differential contributions of the CD4+ and CD8+ compartments among animals.

### Rapid recall and boosting of cellular immune responses in “primed” animals upon challenge with H1N1pdm

By day 28 after infection all 5 animals made antibody responses against the seasonal H1N1 virus K173, but they made no detectable neutralizing antibodies against H1N1pdm (compare **Tables 1** and 2). We therefore next asked whether other immune responses “primed” by K173 infection could affect the course of infection with H1N1pdm. Four months after inoculating the first 5 animals with K173, we challenged them, along with 7 naive control animals, with H1N1pdm isolate A/California/04/2009 (CA04).

**Figure 4:**
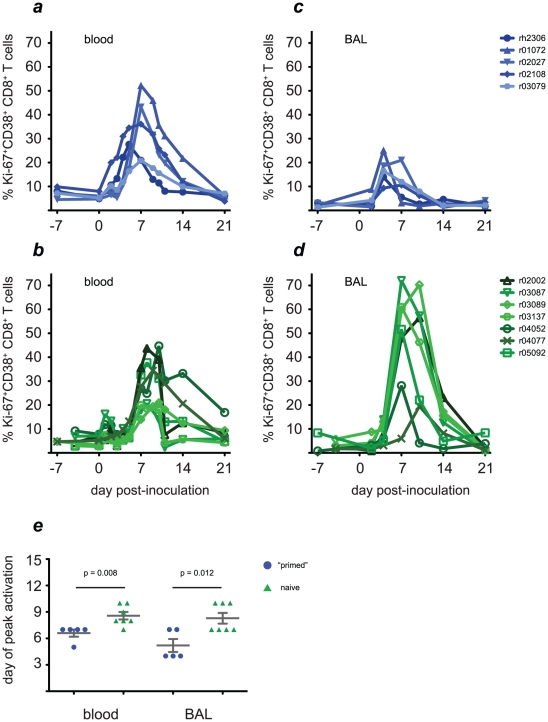
Activated CD8+ T cell populations increase rapidly in the blood and lungs of “primed” animals after challenge with H1N1pdm. Four months after infection with the K173 seasonal isolate, we challenged the 5 previously infected macaques (blue traces, solid symbols) and 7 naive control animals (green traces, open symbols) with 9 million pfu of H1N1pdm isolate A/California/04/2009 (CA04) and monitored the kinetics of CD8+ T cell activation. **a and b**, CD8+ T cell activation kinetics in blood. **c and d**, CD8+ T cell activation kinetics in lung, assessed using cells recovered from bronchoalveolar lavage (BAL). **e**, Peak levels of activation are reached significantly earlier in both compartments in “primed” animals than in naive animals.

We first assessed the kinetics of T cell activation in blood and lung in both groups of animals. Activated CD8+ T cells were detectable in blood as early as 2 days after inoculation with H1N1pdm in “primed” animals and reached peak levels 5–7 days after infection **(Fig. 4a)**. In contrast, in naive animals expansion of activated CD8+ T cells became detectable 4–7 days after infection and reached peak levels 7–10 days after infection, similar to our observations of CD8+ T cell activation following K173 infection in the “primed” animals (**Fig. 4b**, compare to **Fig. 2**). Strikingly, activated CD8+ T cells appeared in the lungs of all 5 “primed” animals within 4 days of H1N1pdm challenge. Indeed, CD8+ T cell activation peaked on day 4 post-infection in 3 of the 5 “primed” animals, and on day 7 post-infection for the remaining 2 **(Fig. 4c)**. In contrast, the frequency of activated CD8+ T cells did not increase above background levels until day 4 post-infection in naive animals, and the peak of activation was not reached until days 7–10 **(Fig 4d)**. Despite this later initiation of cellular immune responses, the frequency of activated CD8+ T cells in BAL samples at the peak of the response was extremely high in 5 of the 7 naive animals (51.7%–72.1% of all CD8+ T cells) in comparison to the frequencies observed in the other 2 naive animals or the 5 “primed” animals (10.6%–28.1% of all CD8+ T cells). The timing of peak CD8+ T cell activation in both blood and lung occurred significantly earlier in “primed” than in naive animals (p = 0.008 and 0.012, respectively; **Fig. 4e**), suggesting that memory CD8+ T cell responses elicited by K173 were rapidly recalled upon H1N1pdm challenge four months later. Similar to our observations after infection with the seasonal virus K173, the frequency of activated CD3+CD8- cells remained low in the blood of both naive and “primed” animals, rising above background levels only in r01072 among the “primed” macaques and r02002 in the naive group **(Fig. S5)**. In contrast, there were modest increases in activated CD3+CD8- cells in the BAL of “primed” animals on day 4 post-infection (peak frequency 5.2–13.8% of BAL CD3+CD8- lymphocytes), but marked increases in the frequency of cells with this phenotype between days 4 and 14 in naive animals (peak frequency 7.0–63.9% of BAL CD3+CD8- lymphocytes), suggesting that activated CD4+ T cells localized to the lung, but mostly did not traffic through the blood, after H1N1pdm infection **(Fig. S5)**.

**Figure 5:**
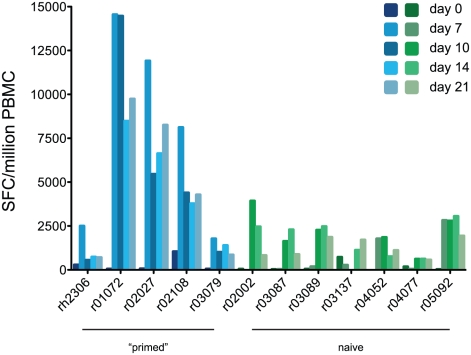
“Primed” animals mount stronger virus-specific T cell responses than naive animals after H1N1pdm challenge. T cell responses against the viral proteome were measured using IFN-γ Elispot as described in Fig. 3. Blue bars, “primed” animals; Green bars, naive animals.

Next we determined whether “priming” of T cell responses by K173 infection affected the magnitude of virus-specific responses following H1N1pdm challenge. Since we were most interested in potentially cross-reactive T cell responses “primed” by K173 infection that could be recalled after H1N1pdm challenge, for these assays we used the same set of overlapping peptides representing the entire influenza virus proteome as we did to measure responses to the initial K173 infection in the “primed” animals, except these assays used peptides representing the CA04 (autologous) HA sequence in order to detect responses against the divergent HA of the challenge virus. As shown in **Fig. 5**, “primed” animals swiftly mounted virus-specific T cell responses upon challenge with H1N1pdm. These responses peaked on day 7 post-infection in all 5 “primed” animals. The peak magnitude of the virus-specific response reached an average of 7,767 SFC/million PBMC in “primed” animals (range 1,770–14,545), an average increase of 3.5-fold per animal (range 1.0–9.7-fold) upon infection with H1N1pdm with respect to K173. In contrast, virus-specific T cell responses peaked on or after day 10 post-infection in all 7 naive animals and reached an average peak magnitude of 2,244 SFC/million PBMC (range 620–3,930). The day on which the peak T cell response occurred was significantly earlier in “primed” than naive animals (p = 0.0035). Interestingly, however, virus-specific T cell frequencies were markedly increased after H1N1pdm infection in only 3 of 5 “primed” animals. Differences in response magnitude were not significant at any point after infection when comparing the “primed” and naive groups as a whole, though they approached significance on day 7 (data not shown). As for K173 infection, assays using CD8+ cell-depleted PBMC showed that peptide-specific responses involved both CD4+ and CD8+ T cells in “primed” and naive animals (data not shown). ICS assays using selected peptide pools suggested that the strongest recall responses in “primed” animals at 7–10 days post-challenge were mediated by CD8+ T cells that secreted IFN-γ but not IL-2 **(Table S3)**. This analysis showed a similar pattern of cellular immune responses to H1N1pdm in naive animals at day 21 post-challenge **(Table S3)**. T cells directed against the N-terminal half of NP were immunodominant in all 5 “primed” animals, similar to our observations after infection with K173, though these responses were co-dominant with populations recognizing the C-terminal half of CA04 HA in r02027 and r03079 **(Fig. S6)**. Naive animals infected with CA04 tended to have more broadly directed T cell responses. Populations recognizing NP peptides were clearly immunodominant in 4 of 7 naive animals, while the other 3 animals' responses were either dominated by different specificities, or were more broadly directed, lacking clearly immunodominant populations **(Fig. S6)**.

**Figure 6:**
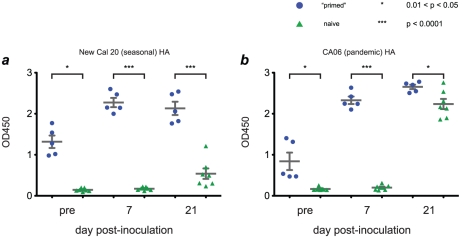
Infection with seasonal influenza viruses induces variable levels of antibody capable of binding H1N1pdm antigens. We used purified hemagglutinin (HA) proteins to capture antibodies from macaque serum. Antibodies were detected using an anti-IgG antibody coupled to horseradish peroxidase, with enzymatic activity quantified using a colorimetric assay. Results are expressed in terms of sample absorbance at 450 nm. Antigens used in each assay were as follows: **a**, Purified HA protein from A/New Caledonia/20/1999, a close antigenic match to K173. **b**, Purified HA from A/California/06/2009, a close antigenic match to CA04. Assays using lysates of K173 or CA04 viruses gave similar results (data not shown).

Neutralizing antibody responses against H1N1pdm were not detectable in either group of animals at day 7 after infection, when both groups of animals appeared to clear infection, although H1N1pdm-specific antibodies were detectable by day 21 **(Table 2)**. To determine whether the animals made antibodies capable of binding, but not neutralizing, H1N1pdm, we performed enzyme-linked immunosorbent assays (ELISAs) using purified HA proteins to capture serum antibodies. These assays showed that at least some antibodies raised by K173 infection were capable of binding a pandemic virus HA (**Fig. 6**). Furthermore, H1N1pdm-HA-binding antibodies reached high titers within 7 days of infection in “primed” animals, while they were not detectable until 21 days after infection in naive animals. Assays using whole lysates of K173 or CA04 viruses gave similar results (data not shown). Together, these data suggest that seasonal influenza virus infection raised antibodies capable of binding, but not neutralizing, H1N1pdm, though the level of these “cross-binding” antibodies varied among “primed” animals.

**Table 2:** Serum antibody titers against pandemic virus CA04.

	CA04-specific HI^a^		
animal	H1N1pdm prechallenge	day 7 post-H1N1pdm	day 21 post-H1N1pdm
rh2306	<8^b^	<10	160
r01072	<8	<10	80
r02027	<8	<10	160
r02108	<8	<10	160
r03079	<8	<10	160
r02002	<8	<10	640
r03087	<8	<10	320
r03089	<8	<10	320
r03137	<8	<10	320
r04052	<8	<10	320
r04077	<8	<10	320
r05092	<8	<10	160

a serum HI titers against pandemic virus A/California/04/2009 ^b^HI assays conducted using turkey red blood cells and animal serum with pandemic virus CA04. Detection limit 8 or 10, depending on lowest serum concentration tested.

Altogether, our results show that animals “primed” to make influenza virus-specific T cell responses by infection with a seasonal human influenza virus mount a stronger cellular immune response upon H1N1pdm challenge, and initiate this response more quickly, than naive animals do. Moreover, H1N1pdm-specific neutralizing antibody responses develop later in infection than T cell responses do in both “primed” and naive animals. However, “primed” animals had varying levels of antibodies capable of binding, but not neutralizing, H1N1pdm at the time of pandemic virus challenge.

### “Primed” animals clear H1N1pdm more rapidly than naive animals

**Figure 7:**
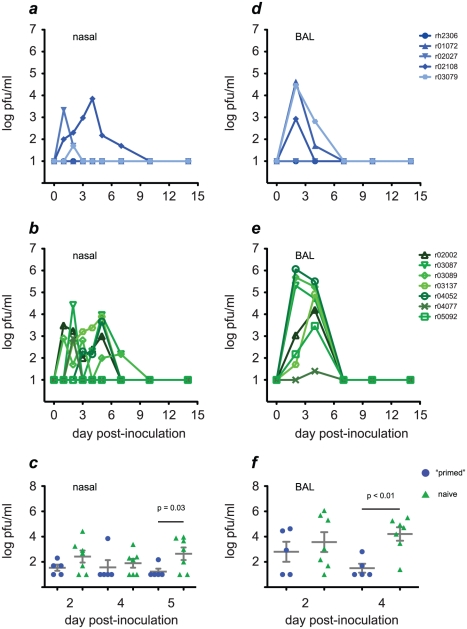
“Primed” animals clear H1N1pdm infection more rapidly than naive animals. We used plaque assays to monitor replication of H1N1pdm in the upper respiratory tract (nasal secretions) and lower respiratory tract (BAL) in “primed” (blue traces, solid symbols) and naive animals (green traces, open symbols). **a and b**, kinetics of H1N1pdm replication in nasal secretions. **c**, By day 5 post-inoculation virus titers in nasal secretions were significantly lower in “primed” than naive animals. **d and e**, H1N1pdm replication kinetics in the lung. **f**, By day 4 post-infection virus titers in lung were significantly lower in “primed” than naive animals.

Our measurements of cellular immune responses in “primed” animals were consistent with what would be expected for an effective cross-reactive memory T cell response, so we next asked whether “primed” animals could clear infection earlier than naive animals could. Indeed, although infectious H1N1pdm was detected in nasal secretions from 6 of 7 naive animals, it was detectable in only 3 of 5 “primed” animals **(Fig 7a and b)**. Moreover, virus titers in the nasal secretions of “primed” animals were significantly lower than those in naive animals by day 5 post-infection, showing that “primed” animals cleared H1N1pdm from the upper respiratory tract earlier than naive animals did **(Fig. 7c)**.

It is thought that robust replication in the lower respiratory tract by avian influenza viruses contributes to their ability to cause severe pneumonia [34], [35]. Since virus replication in the lower respiratory tract may increase the potential for severe disease, we also evaluated H1N1pdm replication in this compartment in both groups of animals. Infectious H1N1pdm was isolated from the lung (BAL fluid) of all 7 naive animals, but from only 3 of 5 “primed” animals **(Fig. 7d and e)**. H1N1pdm titers reached levels above 100,000 pfu/ml BAL fluid by day 2 post-infection in 3 of 7 naive animals, and were 3,000 pfu/ml or greater in 6 of 7 animals on day 4 post-infection. By contrast, H1N1pdm titers never exceeded 100,000 pfu/ml in the lungs of “primed” animals, and infectious virus was detected in only 2 animals (at titers of 650 and 50 pfu/ml) by day 4 post-infection. The difference in lung virus load between “primed” and naive animals on day 4 was significant (p<0.01; **Fig. 7f**). Importantly, H1N1pdm replication was brought under control in the “primed” animals before neutralizing antibodies specific for the challenge virus became detectable, although “primed” animals had varying levels of antibodies capable of binding, but not neutralizing, H1N1pdm at the time of challenge, which reached high titers on day 7. Taken together, our results indicate that previous infection with seasonal influenza viruses can “prime” immunity that enables rapid clearance of challenge with a newly emerging pandemic influenza virus.

## Discussion

It is widely accepted that neutralizing antibody responses are major correlates of protection against influenza viruses; current vaccine guidelines therefore stipulate that a serum antibody titer of ≥1∶40 in a hemagglutination inhibition assay will protect subjects against antigenically matched strains [36]. However, neutralizing antibodies fail to provide broad protection against multiple virus subtypes. In mice T cell responses mediate such “heterosubtypic immunity,” [37] with likely contributions from both CD4+ and CD8+ subsets [38]. T cells could therefore play a particularly important role in protection against newly emerging influenza viruses against which few individuals have effective neutralizing antibodies [39], but studies in inbred laboratory mice could overestimate the capability of T cells to protect against infection. The role of T cells in protecting humans against influenza therefore remains unclear.

Here we used a macaque model to show that prior infection with seasonal human influenza viruses induces strong T cell responses that are rapidly recalled, and in some animals, boosted in magnitude upon challenge with H1N1pdm. Animals “primed” by infection with the seasonal virus cleared H1N1pdm more rapidly from the upper and lower respiratory tract than naive animals, in the absence of pre-existing antibodies capable of neutralizing H1N1pdm. Indeed, serum neutralizing antibodies against H1N1pdm were undetectable in all “primed” and naive animals prior to challenge with the pandemic virus. These observations are consistent with the high degree of sequence divergence between the seasonal H1N1 virus K173 and the pandemic virus CA04 (HA amino acid identity 79.5%, **Table S1**), and also with observations from ferrets and humans that infection with recent seasonal influenza viruses does not appear to raise neutralizing antibodies capable of recognizing 2009 pandemic H1N1 strains [3]–[5], [12]. T cell activation assays suggested that both CD8+ and CD3+CD8- T cells responded to infection with seasonal and pandemic influenza viruses in all “primed” animals. Follow-up Elispot and ICS assays using selected peptide pools agreed with these results.

Taken together, our results suggest that cross-reactive T cell responses may play a key role in facilitating rapid clearance of H1N1pdm in macaques. We speculate that cross-reactive CD8+ T cells are most likely to be involved in killing infected cells, and therefore may be the primary effector of early virus clearance in “primed” animals. However, cross-reactive CD4+ T cell responses also likely provide several functions that serve to augment heterosubtypic immunity, by providing “help” to CD8+ T cells and B cells, and perhaps most importantly by recruiting immune cells to sites of virus replication [40].

There are important caveats to this interpretation of our results. First, we detected antibodies capable of binding H1N1pdm HA protein in “primed” animals at the time of CA04 challenge. The role of such binding, but not neutralizing, antibodies in protection against influenza remains unclear. A mouse study suggested that antibody-mediated functions in addition to direct neutralization of virions can contribute to virus clearance [41]. However, during the 2009 pandemic human subjects with high levels of pre-existing antibodies capable of binding H1N1pdm, but low or undetectable neutralizing antibody titers, were at high risk for severe influenza complications, including lung damage associated with antibody-mediated complement deposition in the respiratory tract [42]. In human H1N1pdm infections, binding, non-neutralizing antibodies therefore appear to be associated more with pathology than with protection. Nonetheless, we cannot exclude the possibility that such antibodies contributed to clearance of H1N1pdm in “primed” macaques.

Second, there was not a clear relationship between the magnitude of T cell responses assessed by activation or Elispot assays and levels of virus replication. Frequencies of activated T cells in the lungs of naive animals were much higher than those in “primed” animals after infection with H1N1pdm, although the frequencies of activated cells in blood were comparable in both groups. T cell responses in blood detected by IFN-γ Elispot were also markedly higher in 3 “primed” animals (r01072, r02027 and r02108) than in all other animals following H1N1pdm challenge. These same 3 animals also had the highest peak levels of CD8+ T cell activation in blood among the “primed” animals, and the highest H1N1pdm virus titers in nasal secretions and/or BAL fluid among the “primed” animals. On the other hand, there was no direct relationship between the magnitude of T cell responses detected by activation assays and Elispot in naive animals. These observations could indicate that our peptide set is more likely to detect cross-reactive recall responses “primed” by the epitope sequences found in K173 than de novo responses to H1N1pdm-derived epitopes. It is possible that sustained high virus titers in the lungs of naive animals drove localized T cell proliferation [43], [44], resulting in strong responses in the lungs that were not yet detectable in the periphery at our latest timepoint. Conversely, early clearance of virus from the lungs of “primed” macaques could have stunted this localized expansion of T cells. Accordingly, the relatively high H1N1pdm titers in r01072, r02027 and r02108 could have driven greater expansion of virus-specific T cells in these animals than in the other “primed” animals. Alternatively, expanding populations of activated T cells in naive animals may be functionally impaired and less able to secrete IFN-γ in response to peptide stimulation than T cells in “primed” animals who more effectively control virus replication. Nonspecific “bystander” activation in the setting of high antigenic load may also contribute to the differences in frequency between T cells with an activated phenotype and cells that secrete cytokines in response to peptide stimulation.

These caveats show that we have not yet fully elucidated the mechanisms of cross-reactive immunity to influenza viruses in macaques. Nonetheless our study establishes an important role for T cells in limiting replication of H1N1pdm in these animals. Future experiments in this system should take advantage of the ability to specifically deplete immune cell subsets (CD4+ cells, CD8+ cells, CD20+ cells, among others) to dissect the contributions of these various populations to immune protection conferred by vaccination and prior infection.

Macaques are increasingly being used to model disease processes caused by pathogenic influenza viruses [21], [23], [29], [31], [45], and to evaluate new vaccine modalities [22], [24], [30], [46]-[57]. Many of these vaccine candidates, for example recombinant virus vectors, are well suited for the induction of CD4+ and CD8+ T cell responses, but so far most studies have focused on developing vectors expressing HA genes only [24], [47], [54]. Perhaps as a result, the majority of macaque studies have examined humoral responses to infection and/or vaccination. Various adjuvanted vaccine preparations [49], [51]–[53], a cold-adapted H5N1 virus [22], and DNA vaccines [30], [51], [55] have all been reported to induce cellular immunity to influenza antigens in macaques, but the contribution of these responses to protection from challenge has only rarely been directly evaluated.

Interestingly, one of these studies showed that vaccination with plasmid DNA encoding only a consensus H5N1 nucleoprotein sequence induced T cell responses capable of limiting replication of a virulent H5N1 challenge, resulting in a 2-log reduction in tracheal virus titers in comparison to naive animals by 6 days post-infection [30]. Vaccines that included both NP and consensus H5N1 HA sequences, and thus induced both T cells and neutralizing antibody, were still more effective, reducing titers by up to 5 logs by day 6. Here, we observed a significant 3–4-log reduction in H1N1pdm titers in “primed” animals by day 4 or 5 post-infection in lower or upper respiratory tract respectively. Laddy et al. did not measure T cell responses after H5N1 virus challenge, so we cannot compare recall responses in their study to those observed here, but the magnitude of T cell responses after the K173 virus “prime” in our animals (average 2,202 SFC/million PBMC; range 1,495–3,745) was similar to the magnitudes they observed after DNA vaccination (1,000–2,000 SFC/million). Differences in control of H5N1 and H1N1pdm challenge in these two studies could therefore be due to multiple factors, including immune response magnitude, relative contributions of T cells and antibodies, virus tropism and route and/or dose of inoculation.

It has been difficult to define the role of T cells in human immunity to influenza, since investigators must rely largely on epidemiological data and a small number of in-vivo challenge experiments. There is evidence that T cell responses may have reduced susceptibility to clinically apparent influenza during the 1957 pandemic [14] and reduced disease severity in individuals infected in 2009 [58]. Similarly, pre-existing T cell responses, but not vaccine-induced antibody levels, correlated with reduced risk for influenza disease in a prospective study of elderly subjects [20]. Virus shedding was also significantly reduced in a subset of experimentally infected subjects who had virus-specific cytotoxic T cell activity in the absence of detectable antibody [15]. Finally, several recent studies have reported that human volunteers have T cells capable of recognizing peptides derived from H5N1 avian viruses and H1N1pdm, and/or peptide epitopes that are conserved among these viruses and seasonal strains [16]–[19], [59]–[61]. Together, these studies show that humans are capable of making cross-reactive T cell responses against influenza viruses, though the role of these responses in protecting subjects from infection or disease remains unclear.

Our results are congruent with these more circumstantial data in humans, and consistent with the close phylogenetic relationship between macaques and humans. Indeed, the magnitude and kinetics of influenza virus replication in our animals closely resembled those observed in experimentally inoculated humans [32]. However one must remember that every animal model has limitations, and even macaque experiments are not always predictive of results in humans. For example, here we waited 4 months between the initial infection with seasonal viruses and rechallenge with H1N1pdm. This is longer than the 4–8 weeks that is typical for evaluations of heterosubtypic immunity in small animals, but the interval between human influenza seasons (and between pandemics) is of course longer still. In mice it appears that the half-life of effector T cell responses, particularly in the respiratory mucosa, is less than one year [62], suggesting that the T cell responses observed in our study could wane below protective levels in the interval between influenza seasons in naturally infected or vaccinated humans. The rates at which mucosal T cell responses decay in humans and primates are not well understood, so this will be an important focus of future studies. Furthermore, there may be important differences in T cell immunobiology between macaques and humans that further limit the translatability of our results.

Finally, our results shed new light on the potential goals of a broadly reactive influenza vaccine. Recent studies have shown that trivalent inactivated vaccines (TIVs) elicit T cell responses in a minority of healthy adults [63], while live attenuated influenza vaccines (LAIVs) can elicit higher-frequency T cells in more subjects [64]. In contrast, a different group showed that LAIV elicited CD8+ T cell responses in children, but neither TIVs nor LAIVs stimulated cellular immunity in adults [65], while Co et al. found that commercial vaccine formulations differ in their ability to stimulate T cell responses in adult-derived cell lines [66]. Taken together, these results show that current vaccine formulations are not consistently immunogenic for T cell responses in humans, and are therefore unlikely to provide heterosubtypic protection against emerging virus variants. In contrast, the rapid clearance of virus observed in our “primed” animals may be sufficient to prevent severe disease and death in the case of a rapidly spreading pandemic caused by a serologically novel virus, which must be the goal of any broadly protective vaccine.

We therefore propose that vaccines capable of consistently inducing strong cross-reactive T cell responses (and/or of boosting pre-existing ones) could provide an important measure of protection against future influenza pandemics. The experiments described here used a seasonal H1N1 virus to “prime” responses for an H1N1pdm challenge, but in principle T cell responses could cross-react with a broad range of isolates and subtypes, provided that the specific epitopes they target are preserved among viruses. In practice, sequences encoding T cell epitopes are likely to be differentially conserved, suggesting that immunogens for future T-cell-based vaccines will need to be chosen carefully [67]. Effective induction of T cell responses would likely require vaccine components that express viral antigens de novo in targeted cells, such as plasmid DNA, live attenuated influenza viruses, or recombinant vectors based on other viruses. Other than live attenuated viruses, such modalities have not been widely tested as influenza vaccines in humans or primates, but recent results appear promising. For example, a replication-defective vaccinia virus vector encoding influenza virus NP and matrix proteins was shown to be safe and capable of inducing T cell responses in humans; the efficacy of this vaccine candidate is currently being evaluated [68]. Our results suggest that vaccine components optimized to induce cross-reactive T cell responses could enhance existing, antibody-based modalities by eliciting cross-reactive T cell responses capable of blunting the replication of even divergent influenza viruses.

## Materials and Methods

### Research animals and ethics statement

This study used rhesus macaques (Macaca mulatta) of Indian descent. Animals were males and females (6 of each gender) between the ages of 4 and 8 (average age 6.17 years). Experiments were performed using the same stocks of seasonal and pandemic viruses in 3 different waves in 2009 and 2010, with slightly different sampling schedules. The study was conducted according to the guidelines of the United States National Research Council [69] and the Weatherall Report [70] under a protocol approved by the University of Wisconsin Graduate School Animal Care and Use Committee. All procedures (virus inoculations, blood draws, bronchoalveolar lavages) were performed under ketamine or ketamine/medetomidine anesthesia, and all efforts were made to minimize suffering. Prior to inclusion in the study animals were screened for the presence of common MHC class I alleles by PCR using sequence-specific primers (PCR-SSP) as previously described [71]. All animals chosen for this study expressed the high-frequency allele Mamu-A*01.

### Viruses and inoculations

A stock of the seasonal human influenza virus isolate A/Kawasaki/173/2001 (H1N1) was produced on Madin-Darby canine kidney (MDCK) cells using reverse genetics, as previously described [72]. A/California/04/2009 (H1N1pdm) was isolated on MDCK cells. Animals were inoculated with a total of 9 million pfu virus via a combination of routes, as described previously [31]. In this method, virus is applied to the trachea (4 ml), tonsils (0.5 ml) and conjunctivae (0.5 ml). Virus titer in nasal and tracheal secretions as well as bronchoalveolar lavage (BAL) fluid was determined using standard plaque assays performed in duplicate on MDCK cells.

### T cell activation assay

We measured activation of CD8+ T cells using an adaptation of a previously described method [33]. Briefly, 100 µl whole blood was stained with CD3 Pacific Blue clone SP34–2 (BD Biosciences, San Jose, CA), CD38 FITC clone AT-1 (Stem Cell Technologies, Vancouver, Canada), CD95 PE-Cy7 clone DX2 (eBioscience, San Diego, CA), CD20 AF700 clone 2H7 (BioLegend, San Diego, CA), and CD8 PerCP clone SK1 (BD Biosciences, San Jose, CA) or CD8 APC-Cy7 clone RPA-T8 (BioLegend, San Diego, CA) for 30 minutes at room temperature in the dark. One ml FACSlyse (BD Biosciences, San Jose, CA) was then added and incubated for 10 minutes at room temperature. The cells were then centrifuged and washed twice with FACS buffer. After washes were complete, 100 µl FACS buffer containing 0.1% saponin was added to permeabilize the cells, which were then stained intracellularly with Ki-67 AF647 clone B56 (BD Biosciences, San Jose, CA), incubated for 30 minutes at room temperature and washed twice with FACS buffer. Alternately cells obtained from BAL fluid were treated in the same fashion as whole blood but no FACSlyse was used; instead cells were fixed in 1% paraformaldehyde for 15 minutes at room temperature. Events were collected on a BD LSRII (BD Biosciences, San Jose, CA) and analyzed using FlowJo software (Tree Star Inc., Ashland, OR).

### IFN-γ Elispot assay

PBMC were separated from whole EDTA-treated blood by Ficoll-Paque PLUS (GE Health Sciences) density centrifugation. The PBMC were used directly in precoated ELISpot^PLUS^ kits (MABTECH Inc., Mariemont, OH) for the detection of monkey IFN-γ according to the manufacturer's protocols. Briefly, 1.0×10^5^ PBMC were used per well and incubated with pools of overlapping 17-mer peptides for approximately 18 hours at 37°C in 5% CO_2_. 19 peptide pools collectively represented the amino acid sequences of all known influenza proteins except PB1F2. Peptides in pools were diluted to a final concentration of 1 µM each. Each plate contained a negative (no peptide) and positive (concanavalin A) control. Results are expressed as the average number of spot-forming cells (SFC) per million PBMC detected for each pool, with the background (average number of SFC/million in negative control wells) subtracted. Peptides representing HA sequences of autologous H1N1pdm isolate CA04 were included in these experiments after H1N1pdm infection. Experiments were conducted in duplicate for each of 19 pools; the coefficient of variance of results per pool ranged between 0 and 71%. Peptides were obtained from BEI Resources (Manassas, VA). Results were considered positive if wells contained an average of ≥5 spots and the average SFC/million detected in peptide-containing wells was at least threefold higher than the average number of spots in negative control wells. The percent amino acid identity between the peptide libraries used in Elispot assays and the challenge viruses K173 and CA04 is shown in Table S1.

### Intracellullar cytokine staining (ICS)

Previously isolated PBMC were thawed and placed in complete RPMI-1640 containing 10% fetal calf serum (R10) and then centrifuged to remove residual freezing media. PBMC were then resuspended at 2x10^7^ cells/ml and aliquoted into 1.2 ml cluster tubes at 1 million PBMC per tube. The following was then added to each tube: 1 µl anti-CD28 clone L293 (BD Biosciences, San Jose, CA), 1 µl anti-CD49d clone 9F10 (BD Biosciences, San Jose, CA), 2 µg brefeldin A (to halt protein transport) and 44 µl R10. Next, peptide pools were added to each tube to a final concentration of 1 µM per peptide. Phorbol 12-myristate 13-acetate (PMA) at 100 ng/ml and ionomycin at 2 µg/ml was added to one tube of PBMC from each animal as a positive control. Tubes were then incubated at 37°C at 5% CO_2_ for six hours. When incubation was complete, LIVE/DEAD violet viability stain (Invitrogen, Carlsbad,CA) was added according to the manufacturer's instructions and the following surface stain antibodies were added: CD3 PerCP-Cy5.5 clone SP34–2 (BD Biosciences, San Jose, CA), CD8 APC-Cy7 clone RPA-T8 (BioLegend, San Diego, CA), and CD4 PE-Cy7 clone OKT4 (BioLegend, San Diego, CA). Cells were then incubated for 30 minutes at room temperature in the dark. Afterwards, cells were washed twice with FACS buffer and fixed in 1% paraformaldehyde for 15 minutes. When fixation was complete, cells were washed once in FACS buffer and left overnight at 4°C. The following day cells were washed twice with FACS buffer containing 0.1% saponin and then stained with the intracellular antibodies IFN-γ FITC clone 4S.B3 (BD Biosciences, San Jose, CA) and IL-2 PE clone MQ1-17H12 (BD Biosciences, San Jose, CA) for 50 minutes at room temperature in the dark. After staining was complete cells were again washed twice with FACS buffer containing 0.1% saponin and stored at 4°C. Events were collected on a BD LSRII flow cytometer and analyzed using FlowJo software.

### Hemagglutination-inhibition (HI) assay

HI assays were performed using turkey red blood cells as previously described [73]. Results are expressed as the reciprocal serum titer at which inhibition of hemagglutination by the indicated virus was no longer observed.

### Antigen preparation

To prepare whole virus lysates for antibody-capture ELISAs, supernatant containing virus grown on MDCK cells was overlaid on a 25% sucrose cushion and spun at 75,000 x g for 2 hours at 4°C. When the spin was completed the supernatant was removed and the virus pellet was resuspended overnight at 4°C in 50 mM Tris-HCl (pH 7.8) containing 0.5% Triton X-100. Purified HA proteins expressed in 293 cells were purchased from Immune Technology Corp. (New York, NY) for use in ELISAs.

### Enzyme linked immunosorbent assay

We used antibody capture ELISA to detect antibodies in macaque serum capable of binding influenza virus antigens, regardless of their ability to neutralize viral infectivity. Briefly, Immunlon 2HB plates (Thermo Fisher Scientific, Waltham, MA) were coated with approximately 1 µg antigen (purified HA protein or whole virus) in PBS at 4°C overnight. Antigen was removed and plates were washed six times with PBS + 0.05% Tween-20. Plasma was diluted 1∶100 in PBS and 100 µl was added to each well. Plates were incubated with diluted plasma at 37°C for 2 hours and then washed six times with PBS + 0.05% Tween-20. To detect bound macaque antibody, mouse anti-human IgG antibody clone G18–145 conjugated to horseradish peroxidase (BD Biosciences, San Jose, CA) was diluted 1∶1000 in PBS and 100 µl were added to each well and incubated at room temperature for one hour. The plate was then washed six times with PBS + 0.05% Tween-20 and SureBlue TMB Microwell Peroxidase substrate (KPL, Gaithersburg, MD) was added and incubated at room temperature. When a blue color change was present in control wells 100 µl 1N HCl was added to each well to stop the reaction. Absorbance at was measured at 450 nm.

### Statistical analysis

Analyses of the differences in timing of peak CD8+ T cell activation, and in the magnitude of peptide-specific T cell responses, virus titer and binding antibody responses in “primed” and naive animals were performed using the Welch-corrected two-tailed t test in Prism software version 5.0c (GraphPad Software, La Jolla, CA). Analyses of virus load were performed on log-transformed data. Analysis of the differences in timing of peak T cell response magnitude detected by Elispot were conducted using the Wilcoxon rank sum test, since the peak occurred on the same day for all “primed” animals.

### Accession numbers for genes described in this study

The 2009 H1N1 pandemic influenza virus strain used to challenge macaques was A/California/04/2009, whose genomic RNAs have the following Genbank accession numbers: FJ969516 (PB2), GQ377049 (PB1), FJ969515 (PA), GQ117044 (HA), FJ969512 (NP), FJ969517 (NA), FJ969513 (M), FJ969514 (NS). The antibody ELISA assays used purified HA subtype 1 proteins from the viruses A/New Caledonia/20/1999 (Genbank CY033622) and A/California/06/2009 (Genbank FJ966960). Sequences for virus A/Kawasaki/173/2001 are not currently available in Genbank.

## Supporting Information

Figure S1 **HLA-DR is not consistently upregulated on activated rhesus macaque T cells.** In our initial experiments we stained lymphocytes in fresh whole blood for a combination of markers associated with activated, proliferating T cells, including Ki-67, CD38, HLA-DR and Bcl-2. (The latter is thought to be downregulated in activated T cells, while the rest are upregulated.) However, we chose not to include HLA-DR staining in the analyses presented in our manuscript, because we found that many animals had relatively high levels of HLA-DRdim CD8+ T cells prior to infection, making a clear definition of HLA-DR+ cells difficult (data not shown). Furthermore, HLA-DR did not appear to be consistently upregulated on CD8+ T cells that otherwise showed the expected upregulation of markers associated with activation. For example, at day 7 after infection, when activated CD8+ T cell frequencies are typically high, animal r04052 shows relatively dim staining with HLA-DR, but robust upregulation of Ki-67 and CD38 (panel **a**). This is not due to a lack of HLA-DR-specific antibody in the sample (far right). In contrast, HLA-DR expression is clearly upregulated on activated CD8+ T cells in animal r02108 at the same timepoint (panel **b**). Since HLA-DR is known to be upregulated on activated human CD8+ T cells, we are currently determining whether different HLA-DR-specific antibody clones give clearer results in our assays. Unfortunately, however, we will not be able to retrospectively analyze PBMC from animals in these studies, since cryopreserved PBMC have already been used for other assays.
(EPS)

Figure S2 **CD3+CD8- cell frequencies approximate those of CD3+CD4+ cells in T cell activation assay.** Our initial experiments used fresh PBMC and BAL cells and stained for a combination of 8 markers that included CD3, CD8, CD38, Ki-67 and HLA-DR, but did not include CD4. Unfortunately, due to constraints in blood draw volume and cell yields, no BAL cells remain; we were, however, able to cryopreserve PBMC from some timepoints. To determine whether frequencies of activated (Ki-67+ CD38+) CD3+CD8-negative T cells measured in our initial experiments accurately estimated frequencies of activated CD4+ T cells, we thawed PBMC taken at day 10 post-infection with the seasonal virus A/Kawasaki/173/2001 (K173; panels **a-d**) or the pandemic virus A/California/04/2009 (H1N1pdm; panels **e-g**) and stained them with antibodies specific for CD3, CD4, CD8, CD38, Ki-67 and HLA-DR. **a**, initial results from fresh PBMC without direct CD4 staining. **b**, We compared the frequency of activated CD4+ T cells measured in cryopreserved day 10 samples with direct CD4 staining (blue squares) to frequencies estimated by gating on CD3+CD8-negative events in freshly stained samples (green circles). **c and d**, As a control for potential changes in lymphocyte quality after freezing and thawing, we compared the frequency of activated CD8+ T cells in fresh and frozen samples. Note that cryopreserved PBMC were not available for animal r02108. We performed a similar analysis of activated CD4+ T cell frequency after H1N1pdm infection in both previously infected (**e**) and naive animals (**f**). **g**, comparison of the frequency of activated CD4+ T cells measured in cryopreserved day 10 samples with direct CD4 staining (blue squares) to frequencies estimated by gating on CD3+CD8-negative events in freshly stained samples (green circles). Frequencies of activated CD4+ T cells measured directly in cryopreserved samples generally agreed with frequencies estimated by gating on CD3+CD8-negative populations in assays of lymphocytes in freshly obtained whole blood. These results indicate that we are unlikely to have missed high-frequency populations of activated CD4+ T cells using our technique.
(EPS)

Figure S3 **Activated CD8- T cells appear in blood and lung within 7 days of inoculation with seasonal influenza virus K173.** We did not directly stain for CD4 in the T cell activation experiments. However, since we did stain for both CD3 (a marker of all T cells) and CD8, we could use the same flow cytometric assay described in Fig. 2 to enumerate CD8- T cells with an activated phenotype, and thus estimate the frequency of activated CD4+ T cells. Data plotted are the frequency of CD3+CD8- lymphocytes that express both Ki-67 and CD38. Data are not available from animal r01072 at day 21 post-inoculation due to technical problems.
(EPS)

Figure S4 **T cells recognizing NP antigens are immunodominant in macaques infected with a seasonal H1N1 influenza virus.** We assessed the ability of 19 different pools of synthetic peptides representing influenza virus antigens to stimulate secretion of IFN-γ in PBMC from macaques infected with the seasonal human virus K173. Peptides representing most viral proteins were divided into 2 pools, with pool A containing peptides from the N-terminal half and pool B containing peptides from the C-terminal half. Responses directed against, e.g., NP-A therefore recognize epitopes in the N-terminal half of the protein. Colors indicate the proportion of the total virus-specific response recognizing various peptide pools. The 4 peptide pools targeted by the highest-frequency T cells in each animal are shown. Responses to all other pools are combined and shown in grey. Note that the pools shown differ slightly among animals, as do vertical scales.
(EPS)

Figure S5 **Activated CD8- T cells reach lower peak frequencies and peak earlier in “primed” than in naive animals after challenge with H1N1pdm.** We estimated the frequency of activated CD4+ T cells in blood and lung of “primed” and naive animals after inoculation with H1N1pdm isolate A/California/04/2009 as in Fig. S3. “Primed” animals are shown using blue traces and solid symbols. Naive animals are shown using green traces and open symbols. Data are not available from animal r01072 at day 21 post-inoculation due to technical problems. **a and b**, CD3+CD8- T cell activation kinetics in blood. **c and d**, CD3+CD8- T cell activation kinetics in lung, assessed using cells recovered from bronchoalveolar lavage (BAL).
(EPS)

Figure S6 **T cells recognizing NP antigens are immunodominant in “primed” macaques after challenge with H1N1pdm.** We assessed the ability of 19 different pools of synthetic peptides representing influenza virus antigens to stimulate secretion of IFN-γ in PBMC from macaques infected with the pandemic human virus CA04. Peptide pools were constructed, and data is displayed, as described for Fig. S2, with the addition of pools specific for the HA of CA04. The 4–6 peptide pools targeted by the highest-frequency T cells in each animal are shown. Responses to all other pools are combined and shown in grey. Note that the pools shown differ slightly among animals, as do vertical scales.
(EPS)

Table S1 **Amino acid identity between peptide library viruses and challenge viruses used in this experiment.** This table depicts the % amino acid identity between challenge viruses and synthetic peptide libraries used in this experiment. Pairwise comparisons of challenge viruses to peptide libraries for each viral protein are shown.
(PDF)

Table S2 **Frequencies of influenza virus peptide-specific CD4+ and CD8+ T cells detected by intracellular cytokine staining (ICS) after seasonal virus infection.** This table depicts the frequencies of CD4+ and CD8+ T cells secreting cytokines in response to stimulation with selected peptides. ICS assays were performed on cryopreserved peripheral blood mononuclear cells (PBMC) sampled after infection with the seasonal influenza virus A/Kawasaki/173/2001 (H1N1).
(PDF)

Table S3 **Frequencies of influenza virus peptide-specific CD4+ and CD8+ T cells detected by intracellular cytokine staining (ICS) after H1N1pdm challenge.** This table depicts the frequencies of CD4+ and CD8+ T cells secreting cytokines in response to stimulation with selected peptides. ICS assays were performed on cryopreserved peripheral blood mononuclear cells (PBMC) sampled after infection with the pandemic influenza virus A/California/04/2009 (H1N1).
(PDF)
