# Supplementary material for: Cross-Reactive T Cells Are Involved in Rapid Clearance of 2009 Pandemic H1N1 Influenza Virus in Nonhuman Primates
Source: PLoS Pathog. 2011 Nov 10;7(11):e1002381. doi: 10.1371/journal.ppat.1002381 (PMC3213121; doi:10.1371/journal.ppat.1002381)
Supplement: Table S1 — Amino acid identity between peptide library viruses and challenge viruses used in this experiment. This table depicts the % amino acid identity between challenge viruses and synthetic peptide libraries used in this experiment. Pairwise comparisons of challenge viruses to peptide libraries for each viral protein are shown. (PDF) [file ppat.1002381.s007.pdf]

Table S1. Percent amino acid identity between peptide library viruses and challenge viruses used in this experiment.

protein	peptide source virus[a]	A/California/04/2009	A/Kawasaki/173/2001
HA	A/New Caledonia/20/1999	79.8	99.3
HA	A/California/04/2009	100	79.8
NA	A/New Caledonia/20/1999	81.7	99.2
M1	A/New York/348/2003	93.7	99.7
M2	A/New York/348/2003	82.5	98
NP	A/New York/348/2003	89.8	99.6
NS1	A/New York/348/2003	80.5	99.1
NS2	A/New York/348/2003	86.8	100
PA	A/New York/348/2003	94	99.3
PB1	A/New York/348/2003	94.5	99.4
PB2	A/New York/348/2003	93.9	99

[a]Peptide libraries were constructed using the protein sequences of A/New Caledonia/20/1999 or A/California/04/2009 (H1N1pdm) for HA; A/New Caledonia/20/1999 (H1N1) for NA; A/New York 444/2001 (H1N1) for NS1; and A/New York/348/2003 (H1N1) for the remaining proteins. Tabulated here is the percent amino acid identity between proteins of these viruses and the challenge viruses used in these experiments. Note that H1N1pdm HA peptides were only used after challenge with H1N1pdm.
