# Supplementary material for: Cross-Reactive T Cells Are Involved in Rapid Clearance of 2009 Pandemic H1N1 Influenza Virus in Nonhuman Primates
Source: PLoS Pathog. 2011 Nov 10;7(11):e1002381. doi: 10.1371/journal.ppat.1002381 (PMC3213121; doi:10.1371/journal.ppat.1002381)
Supplement: Table S2 — Frequencies of influenza virus peptide-specific CD4+ and CD8+ T cells detected by intracellular cytokine staining (ICS) after seasonal virus infection. This table depicts the frequencies of CD4+ and CD8+ T cells secreting cytokines in response to stimulation with selected peptides. ICS assays were performed on cryopreserved peripheral blood mononuclear cells (PBMC) sampled after infection with the seasonal influenza virus A/Kawasaki/173/2001 (H1N1). (PDF) [file ppat.1002381.s008.pdf]

Table S2. Frequencies of influenza virus peptide-specific CD4+ and CD8+ T cells detected by intracellular cytokine staining (ICS) after seasonal virus infection.

Animal	Days post infection[a]	Peptide pool[b]	T cell subset	IL-2- IFN-γ+[c]	IL-2+ IFN-γ+[d]
rh2306	21 K173	NA-A	CD8+	0.012	0.012
			CD4+	0	0.024
	21 K173	NP-B	CD8+	0.03	0.075
			CD4+	0.008	0.043
r01072	7 K173	HA-A	CD8+	0	0
			CD4+	0	0.005
	7 K173	NP-A	CD8+	0	0
			CD4+	0	0.028
r02027	21 K173	NP-A	CD8+	0	0
			CD4+	0	0
	21 K173	NP-B	CD8+	0.034	0
			CD4+	0	0
r02108	10 K173	NA-A	CD8+	0	0
			CD4+	0	0
	10 K173	NP-A	CD8+	0	0.012
			CD4+	0	0.028
r03079	7 K173	NP-A	CD8+	0	0.015
			CD4+	0.004	0.012
	7 K173	NP-B	CD8+	0	0
			CD4+	0.004	0.004

[a]ICS assays were performed as described in Materials and Methods on cryopreserved PBMC sampled 7, 10 or 21 days after infection with the seasonal virus A/Kawasaki/173/2001 (K173).

[b]Since the number of cryopreserved PBMC was limited for each animal these assays focused on the 2 peptide pools that stimulated the strongest responses from PBMC in Elispot assays. Letters A and B indicate that peptide pools span the N-terminal and C-terminal halves of the designated protein; e.g. NP-A indicates the N-terminal half of nucleoprotein.

[c,d]Frequencies of CD3+ cells expressing CD4 or CD8 and secreting cytokine(s) are shown. Background cytokine secretion, i.e. the frequency of autologous cells producing cytokine(s) in the absence of peptide stimulation, is subtracted from the data shown. ICS assays detected production of both IFN-γ and interleukin (IL)-2. IL-2- IFN-γ+ indicates cells secreting IFN-γ but not IL-2; IL-2+ IFN-γ+ indicates peptide-specific production of both cytokines.
