# Supplementary material for: Cross-Reactive T Cells Are Involved in Rapid Clearance of 2009 Pandemic H1N1 Influenza Virus in Nonhuman Primates
Source: PLoS Pathog. 2011 Nov 10;7(11):e1002381. doi: 10.1371/journal.ppat.1002381 (PMC3213121; doi:10.1371/journal.ppat.1002381)
Supplement: Table S3 — Frequencies of influenza virus peptide-specific CD4+ and CD8+ T cells detected by intracellular cytokine staining (ICS) after H1N1pdm challenge. This table depicts the frequencies of CD4+ and CD8+ T cells secreting cytokines in response to stimulation with selected peptides. ICS assays were performed on cryopreserved peripheral blood mononuclear cells (PBMC) sampled after infection with the pandemic influenza virus A/California/04/2009 (H1N1). (PDF) [file ppat.1002381.s009.pdf]

Table S3. Frequencies of influenza virus peptide-specific CD4+ and CD8+ T cells detected by intracellular cytokine staining (ICS) after H1N1pdm challenge.

“Primed”/Naive	Animal	Days post infection[a]	Peptide pool[b]	T cell subset	IL-2- IFN-γ+[c]	IL-2+ IFN-γ+[d]
Primed	rh2306	7 CA04	NP-A	CD8+	0.311	0.094
				CD4+	0.03	0.048
		7 CA04	PB2-B	CD8+	0.16	0.014
				CD4+	0.009	0
	r01072	10 CA04	NP-A	CD8+	1.77	0.653
				CD4+	0.142	0.106
		10 CA04	NP-B	CD8+	0.081	0
				CD4+	0.078	0.054
	r02027	7 CA04	HA-B CA04	CD8+	0.162	0.019
				CD4+	0	0.003
		7 CA04	NP-A	CD8+	0.414	0.05
				CD4+	0.104	0.044
	r02108	7 CA04	M1-A	CD8+	0.043	0.002
				CD4+	0.094	0.04
		7 CA04	NP-A	CD8+	0.494	0.104
				CD4+	0.286	0.136
	r03079	7 CA04	HA-B CA04	CD8+	0.093	0.007
				CD4+	0.004	0.034
		7 CA04	NP-A	CD8+	0.017	0
				CD4+	0.014	0.015
Naive	r02002	21 CA04	HA-A CA04	CD8+	0.013	0
				CD4+	0	0.014
		21 CA04	NP-A	CD8+	0.108	0.036
				CD4+	0.002	0.046
	r03087	21 CA04	NP-A	CD8+	0.04	0.106
				CD4+	0.007	0.026
		21 CA04	NS1-B	CD8+	0.168	0.093
				CD4+	0.02	0.011
	r03089	21 CA04	HA-A CA04	CD8+	0.021	0
				CD4+	0.01	0.005
		21 CA04	NP-A	CD8+	0.098	0.044
				CD4+	0	0.02
	r03137	21 CA04	HA-A CA04	CD8+	0	0
				CD4+	0.01	0.006
		21 CA04	NP-A	CD8+	0	0
				CD4+	0.011	0.003
	r04052	21 CA04	HA-B CA04	CD8+	0.047	0.008
				CD4+	0	0
		21 CA04	NP-A	CD8+	0.015	0
				CD4+	0.022	0
	r05092	21 CA04	HA-A CA04	CD8+	0.064	0.015
				CD4+	0	0.002
		21 CA04	NP-A	CD8+	0.418	0.223
				CD4+	0	0.008
	r04077	21 CA04	NP-A	CD8+	0	0.038
				CD4+	0.007	0
		21 CA04	NP-B	CD8+	0	0
				CD4+	0	0

[a]ICS assays were performed as described in Materials and Methods on cryopreserved PBMC sampled 7, 10 or 21 days after infection with the pandemic virus A/California/04/2009 (CA04).

[b]As after K173 infection, assays focused on the 2 peptide pools that stimulated the strongest responses from PBMC in Elispot assays. Letters A and B indicate that peptide pools span the N-terminal and C-terminal halves of the designated protein; e.g. NP-A indicates the N-terminal half of nucleoprotein. Elispot assays included peptides representing the HA proteins of both K173 and CA04; the source of peptide pools used for confirmatory ICS assays is indicated.

[c,d]Frequencies of CD3+ cells expressing CD4 or CD8 and secreting cytokine(s) are shown. Background cytokine secretion, i.e. the frequency of autologous cells producing cytokine(s) in the absence of peptide stimulation, is subtracted from the data shown. ICS assays detected production of both IFN-γ and interleukin (IL)-2. IL-2- IFN-γ+ indicates cells secreting IFN-γ but not IL-2; IL-2+ IFN-γ+ indicates peptide-specific production of both cytokines.
